# Lysophosphatidic Acid Enhances Stromal Cell-Directed Angiogenesis 

**DOI:** 10.1371/journal.pone.0082134

**Published:** 2013-12-02

**Authors:** Bernard Y. K. Binder, Claus S. Sondergaard, Jan A. Nolta, J. Kent Leach

**Affiliations:** 1 Department of Biomedical Engineering, University of California Davis, Davis, California, United States of America; 2 Department of Orthopaedic Surgery, School of Medicine, University of California Davis, Davis, California, United States of America; 3 Department of Surgery, Division of Cardiothoracic Surgery, School of Medicine, University of California Davis, Sacramento, California, United States of America; 4 Departments of Hematology/Oncology, Cell Biology and Human Anatomy and Stem Cell Program, School of Medicine, University of California Davis, Sacramento, California, United States of America; Wake Forest Institute for Regenerative Medicine, United States of America

## Abstract

Ischemic diseases such as peripheral vascular disease (PVD) affect more than 15% of the general population and in severe cases result in ulcers, necrosis, and limb loss. While the therapeutic delivery of growth factors to promote angiogenesis has been widely investigated, large-scale implementation is limited by strategies to effectively deliver costly recombinant proteins. Multipotent adipose-derived stromal cells (ASC) and progenitor cells from other tissue compartments secrete bioactive concentrations of angiogenic molecules, making cell-based strategies for *in situ* delivery of angiogenic cytokines an exciting alternative to the use of recombinant proteins. Here, we show that the phospholipid lysophosphatidic acid (LPA) synergistically improves the proangiogenic effects of ASC in ischemia. We found that LPA upregulates angiogenic growth factor production by ASC under two- and three-dimensional *in vitro* models of serum deprivation and hypoxia (SD/H), and that these factors significantly enhance endothelial cell migration. The concurrent delivery of LPA and ASC in fibrin gels significantly improves vascularization in a murine critical hindlimb ischemia model compared to LPA or ASC alone, thus exhibiting the translational potential of this method. Furthermore, these results are achieved using an inexpensive lipid molecule, which is orders-of-magnitude less costly than recombinant growth factors that are under investigation for similar use. Our results demonstrate a novel strategy for enhancing cell-based strategies for therapeutic angiogenesis, with significant applications for treating ischemic diseases.

## Introduction

Peripheral vascular disease (PVD) affects more than 27 million people in Europe and North America and is characterized by obstruction of blood flow to the extremities [1]. Closely linked risk factors include smoking, hypertension, obesity, diabetes, and age, and severe cases lead to limb necrosis and loss [2]. Unfortunately, surgical interventions for acute PVD are invasive and expensive, necessitating the development of other effective treatment options. One such strategy involves the use of angiogenic cytokines such as the potent endothelial cell mitogen vascular endothelial growth factor (VEGF) to promote revascularization *in situ* [3,4]. However, such growth factors can be cost-prohibitive and difficult to release in a controlled spatiotemporal manner, raising concerns about awaking dormant tumor cells and aberrant vessel formation.

The localized delivery of hydrogel-entrapped proangiogenic cells provides an attractive alternative to current methods [5,6] and obviates the need for additional recombinant proteins. For example, bone marrow-derived mesenchymal stromal cells (MSC) encapsulated in alginate beads improve angiogenesis in ischemic mouse limbs [7]. However, further improvements in function were obtained by transducing the cells to express recombinant telomerase and exogenous peptides to elicit paracrine effects [7], presenting major hurdles for clinical implementation. Compared to MSC, adipose-derived stromal cells (ASC) represent a more clinically appealing population because they can be obtained using minimally invasive procedures and with dramatically higher yields [8,9], allowing for their direct use without further *in vitro* expansion. Furthermore, ASC secrete many angiogenic growth factors including VEGF and have been targeted for use in vascular and musculoskeletal regenerative medicine [6,10-12]. 

Lysophosphatidic acid (LPA) is an inexpensive, commercially available glycerophospholipid that signals through multiple G-protein coupled receptors and is naturally found in serum at low micromolar levels [13,14]. LPA has diverse effects on many cell types and regulates processes such as cell survival [15], migration [16], and differentiation [17]. In particular, LPA promotes VEGF secretion by human MSC [18,19]. This effect is enhanced under hypoxia [20-22], making LPA a natural target for stimulating trophic factor secretion and endothelial cell recruitment in ischemic defects. 

We hypothesized that LPA enhances the proangiogenic effects of ASC under ischemia both *in vitro* and *in vivo*. We tested our hypothesis by exposing human ASC to 25 μM LPA under serum deprivation and hypoxia (SD/H) and examining LPA receptor expression and transcriptional activity of angiogenic growth factors. We assessed the functional effects of LPA by measuring endothelial cell migration towards ASC-conditioned media and quantifying VEGF secretion by ASC suspended in fibrin gels with LPA. Finally, we determined the therapeutic relevance of fibrin-entrapped ASC and LPA by quantifying revascularization in a rigorous *in vivo* model of critical hindlimb ischemia.

## Materials and Methods

### 1.1. Cell culture

Human adipose-derived stromal cells from three male donors (28, 39, and 60 years old) were separately isolated from adipose tissue (National Disease Research Interchange, Philadelphia, PA) as previously described [8]. Cells were expanded in growth medium (GM) consisting of minimum essential alpha medium (α-MEM, Invitrogen, Carlsbad, CA) supplemented with 10% fetal bovine serum (FBS, JR Scientific, Woodland, CA) and 1% penicillin-streptomycin (P/S, Mediatech, Manassas, VA). All angiogenic gene expression assays were performed on each donor. Subsequently, ASC from the 39 year old male were chosen as a representative population for continued characterization and *in vivo* implantation. Cells were cultured under standard conditions in a humidified incubator and utilized at passages 4-5. All medium was replaced every 3 days. 

For all experiments examining the effects of SD/H, ASC were seeded on 6-well tissue culture plates at 25,000 cells/cm^2^. After attaching overnight, cells were washed 3x with PBS to eliminate all traces of serum. To simulate ischemia, media was replaced with serum-free GM supplemented with 0.1% (w/v) fatty-acid free BSA, and cells were incubated in hypoxia for 24 h in Heracell 150i tri-gas incubators (Thermo Scientific, Waltham, MA) at 1% oxygen (*n* = 6). ASC were supplemented with LPA (Enzo Life Sciences, Plymouth Meeting, PA) to a final concentration of 25 μM. A subset of cells received the LPA_1/3_ inhibitor Ki16425 (10 μM; Cayman Chemical, Ann Arbor, MI) to abrogate LPA binding. Negative controls for ischemia were cultured for the same duration in 21% O_2_ in GM with full serum.

### 1.2. qPCR analysis of angiogenic cytokine production and LPA receptor expression

Total RNA was collected from ASC exposed to SD/H, with or without LPA and Ki16425 (*n* = 4) using an RNeasy Mini kit (Qiagen, Valencia, CA). 600 ng of total RNA was reverse transcribed with the QuantiTect Reverse Transcription kit (Qiagen). qPCR was performed using a QuantiFast Probe PCR kit (Qiagen) on a Mastercycler realplex2 (Eppendorf, Westbury, NY. Primers and probes for *RPL13* (HS00204173_m1), *VEGFA* (Hs00900055_m1), *FGF2* (Hs00266645_m1), *LPAR1* (Hs00173500_m1)*, LPAR2* (Hs01113287_m1)*, LPAR3* (Hs00173857_m1)*, LPAR4* (Hs00271072_s1), and *LPAR5* (Hs00252675_s1) were purchased from Applied Biosystems (Foster City, CA). Amplification conditions were 95°C for 3 min, followed by 40 cycles at 95°C for 3 s and 60°C for 30 s. Quantitative PCR results were normalized to *RPL13* transcript levels to yield ΔCt, and fold change in expression relative to the housekeeping gene was calculating using 2^-ΔCt^. 

### 1.3. Transwell migration assay

ASC were exposed to SD/H in the presence or absence of LPA and Ki16425. Conditioned media was collected after 24 h and mixed 1:4 with endothelial cell growth media (EGM2, Lonza, Walkersville, MD) supplemented with 10% FBS and 1% P/S, but no VEGF, FGF, or IGF (GF-def EGM2) [23]. 1×10^5^ human endothelial colony forming cells (ECFC, generous gift of Dr. Mervin Yoder, Indiana University) in 300 μL GF-def EGM2 were seeded on 24-well FluoroBlok™ cell culture inserts (3 μM pore size, BD Biosciences, San Jose, CA) coated in 0.1% bovine gelatin, and inserts were placed in 1 mL of conditioned media mixture. Cells were incubated for 24 h, and cell migration across the membrane was quantified by staining with calcein AM and measuring fluorescence on a microplate reader (Synergy HTTR, Wisnooski, VT) according to the manufacturer’s instructions. 

### 1.4. Fibrin gel fabrication

ASC were entrapped in fibrin gels containing 20 mg/mL fibrinogen and 2.3% (w/v) NaCl as previously described [24]. Briefly, a solution of 40 mg/mL fibrinogen and 4.4% (w/v) NaCl containing 25×106 ASC/mL was mixed in equal volume with a solution containing 5 U/mL human thrombin and 40 mM CaCl2 in PBS. 80 μL of mixture was cast into each well of a polydimethylsiloxane (PDMS) mold and allowed to gel for 1 h at 37°C [25]. When appropriate, LPA and Ki16425 were included in the fibrinogen solution to yield a final concentration of 25 μM and 10 μM, respectively. 

### 1.5. Quantification of ASC angiogenic growth factor production

VEGF secretion over 24 h into media by ASC entrapped in fibrin gels in response to SD/H, 25 μM LPA, and 10 μM Ki16425 was measured using a human VEGF ELISA kit according to the manufacturer’s instructions (R&D Systems, Minneapolis, MN). Data were normalized to the quantity of total DNA collected from the cells in each gel using a Quant-iT PicoGreen dsDNA Assay Kit (Invitrogen) (*n* = 4). To further assess angiogenic trophic factor secretion, media was analyzed using a RayBio® Human Angiogenesis Antibody Array 1 (G-series, RayBiotech, Norcross, GA) according to the manufacturer’s instructions.

### 1.6. Critical limb ischemia model

Treatment of experimental animals was in accordance with UC Davis animal care guidelines and all National Institutes of Health animal-handling procedures. The UC Davis IACUC specifically approved the protocol. The hindlimb ischemia protocol was performed largely as previously described [26]. Briefly, 16 week old nonobese diabetic/severe combined immune deficient gamma (NSG, NOD.Cg-Prkdc^scid^ Il2rg^tm1Wjl^/SzJ) mice (Jackson Laboratories – West, Sacramento, CA) were anesthetized and maintained under a 2% isoflurane/O_2_ mixture delivered through a mask. Unilateral hindlimb ischemia was surgically induced by exposing the right femoral artery and vein and ligating the proximal portion of the femoral artery, the distal portion of the saphenous artery and the remaining collateral arteries after dissecting away the femoral nerve. The ligated artery was excised, completely removed from the hind limb, and fibrin gels containing 25 μM LPA, 1x10^6^ ASC, or both 25 μM LPA and 1x10^6^ ASC were positioned towards the proximal ligation site (*n* = 10). Buprenorphine (0.05 mg/kg) was administered for pain relief at the time of surgery and twice daily for the next 3 days, and animals were allowed access to food and water *ad libitum*. 

Animals were euthanized 2 weeks post-surgery, and both the non-operated and treated limbs were removed, fixed in 10% formalin for 72 h, and transferred to 70% ethanol. The quadriceps muscle from each limb was excised distal to the defect site and processed for histology using standard techniques. Paraffin-embedded specimens were sectioned at 5 μM for staining. 

### 1.7. Blood vessel quantification

Large vessel density was quantified in both the uninjured contralateral limb and treated limb for each animal using H&E stained cross-sections at 100x magnification by a blinded observer. Vessels were enumerated from 10 random fields of view per sample by counting circular structures with well-defined lumens containing more than one erythrocyte. The presence of endothelial cells was determined by immunohistochemistry using antibodies for mouse CD31 (ab124432, 1:1000, Abcam, Cambridge, MA) and a rabbit specific HRP/DAB detection kit (ab64261, Abcam). 

### 1.8. Statistical analysis

Data are presented as mean ± standard error unless otherwise stated. Statistical analysis was performed using one-way ANOVA with Tukey’s Multiple Comparison post-test where applicable. *P* values less than 0.05 were considered statistically significant.

## Results

### 1.1. LPA induces VEGF and FGF2 expression through LPA1

**Figure 1:**
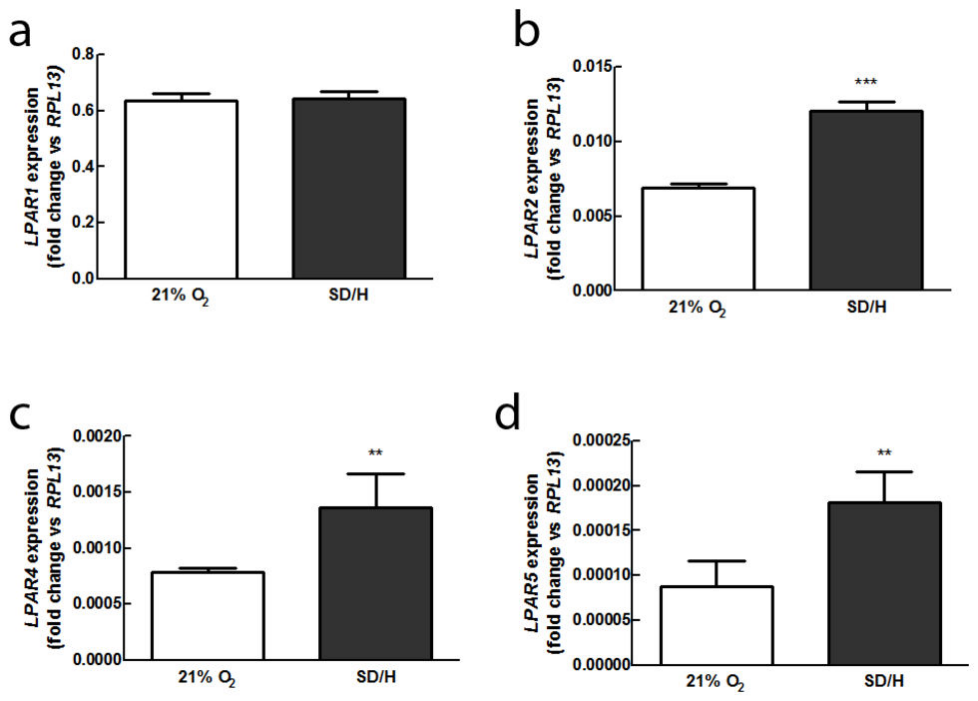
LPA receptor expression in human ASC is dependent on oxygen microenvironment. (**a**) *LPAR1* expression is unchanged by SD/H, but (**b**) *LPAR2* expression is significantly higher in SD/H. *LPAR3* expression is undetectable in either condition, but SD/H also increases expression of (**c**) *LPAR4* and (**d**) *LPAR5* (*n* = 4). **p < 0.01 vs. control, ***p < 0.001 vs. control.

**Figure 2:**
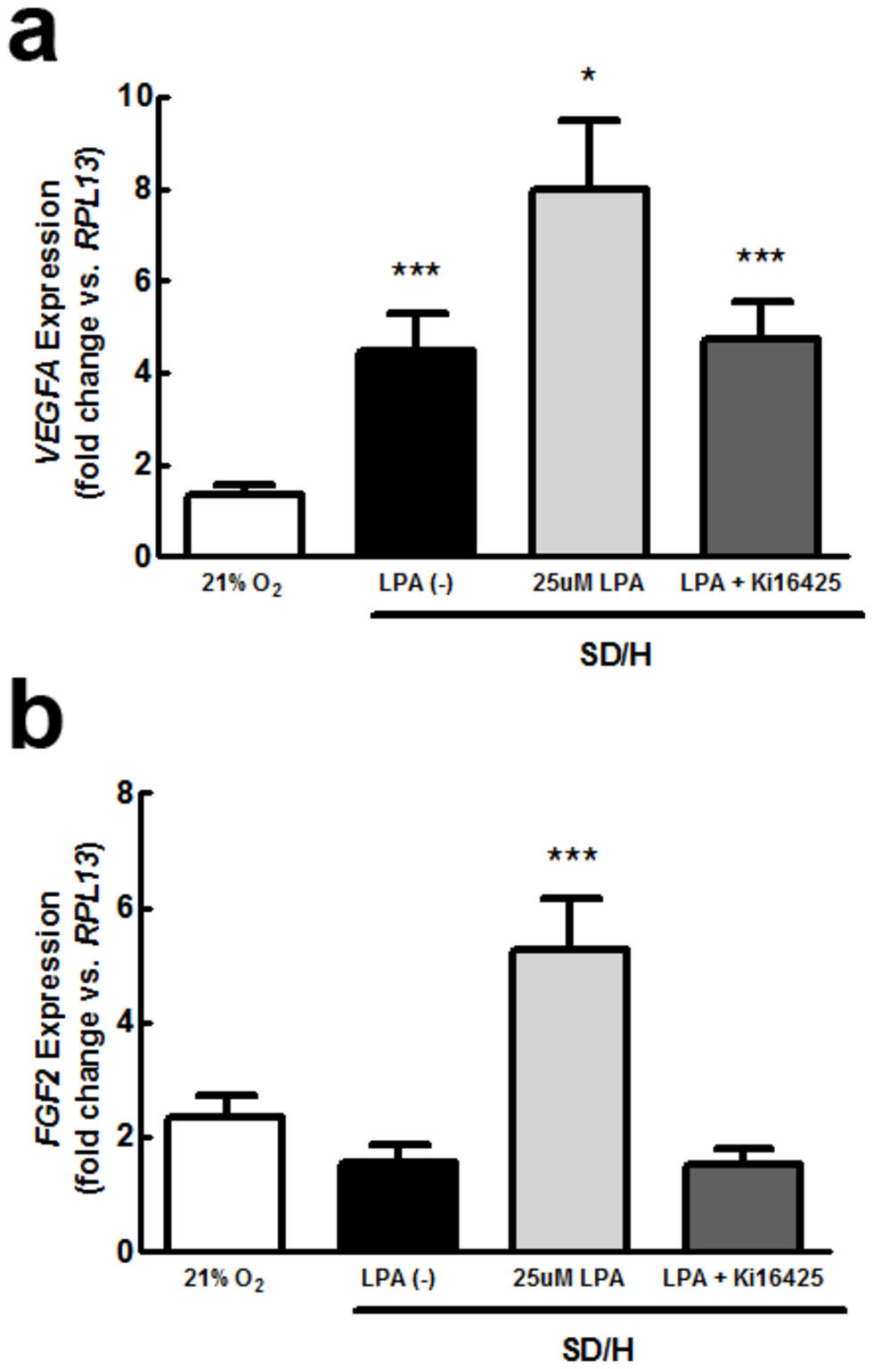
LPA enhances the proangiogenic effects of ASC under ischemia *in* *vitro*. Expression of (**a**) *VEGF* and (**b**) *FGF2* are upregulated by SD/H and further enhanced with the addition of 25 μM LPA. In both cases, the addition of Ki16425 abrogates this effect (*n* = 4). Data represents combined gene expression from three unique donors. *p < 0.05 vs. control, **p < 0.01 vs. control, ***p < 0.001 vs. control.

Since there are multiple receptors for LPA, we assessed gene expression for *LPAR1*, *LPAR2*, *LPAR3*, *LPAR4*, and *LPAR5* in ASC to determine possible signaling pathways. We also compared receptor transcription under standard culture conditions with expression under SD/H to determine whether ischemia might affect LPA sensitivity (Figure **1a-d**). Although *LPAR2*, *LPAR4*, and *LPAR*5 showed increased expression under SD/H, the most prevalent receptor, *LPAR1*, remained unchanged. *LPAR3* was undetected in this cell population. We then compared expression of genes encoding for two potent angiogenic factors, *VEGFA* and basic fibroblast growth factor (*FGF2*) under SD/H in the presence or absence of LPA (Figure **2a-b**). Hypoxia alone induced greater *VEGFA* and *FGF2* transcription compared to normoxia [27], but the addition of 25 μM LPA synergistically increased this effect. When we added the competitive LPA_1/3_ inhibitor Ki16425 to LPA-treated cells, changes in expression were completely abrogated. Thus, based on high expression of *LPAR1* and nearly undetectable levels of *LPAR3*, together with the capacity of the LPA1/3 inhibitor to block the effects of LPA on ASC, we conclude that these effects are mediated through LPA_1_.

### 1.2. ASC treated with LPA promote endothelial cell migration

**Figure 3:**
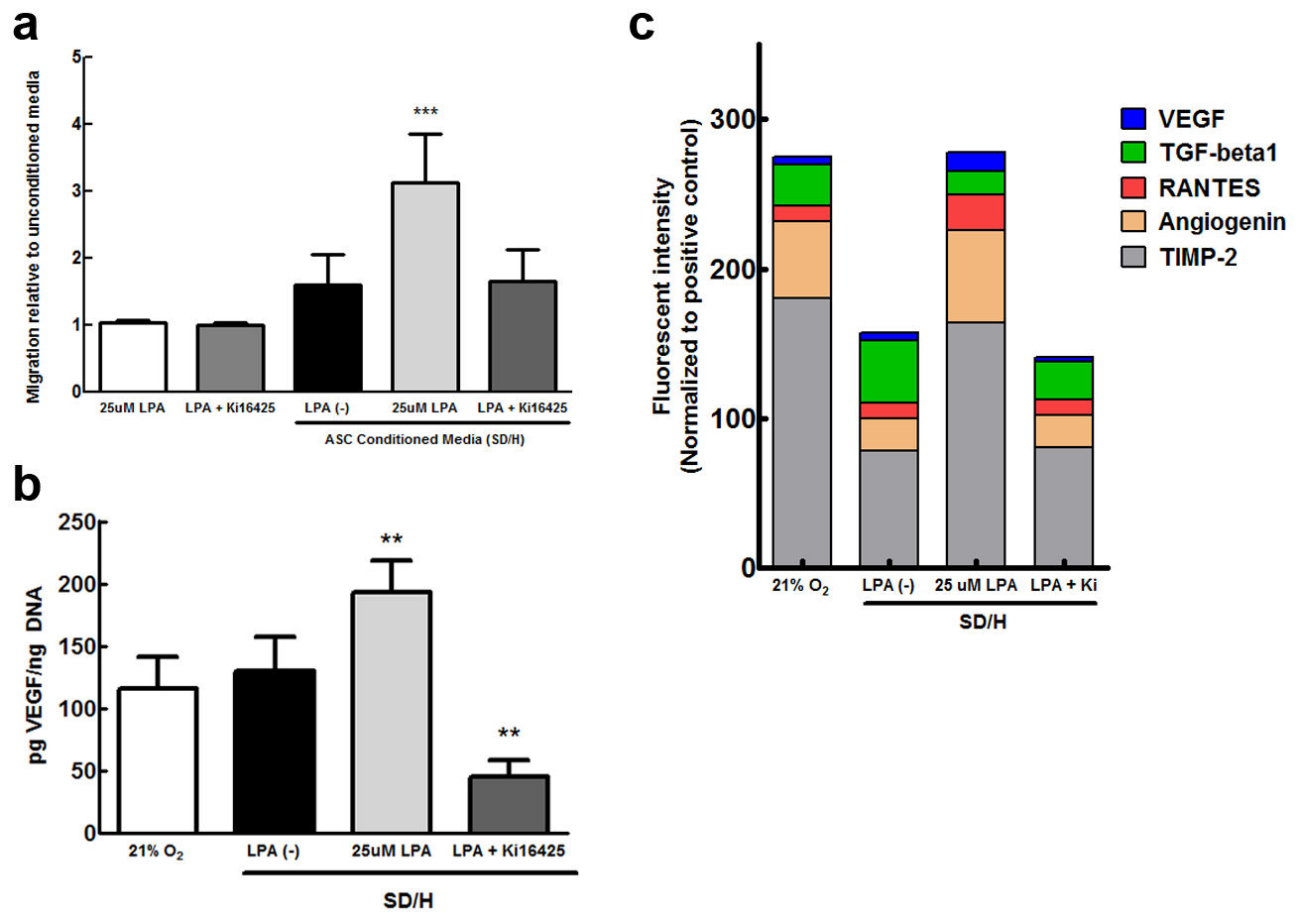
ASC treated with LPA promote endothelial cell migration. (**a**) Medium conditioned by ASC in the presence of LPA under SD/H is significantly more chemoattractive to ECFC than medium from non-treated or inhibitor-treated ASC. The addition of LPA or Ki16425 to unconditioned medium had no effect on ECFC migration (*n* = 5). (**b**) LPA promotes VEGF secretion from ASC entrapped in 3D fibrin gels under SD/H compared to cells treated with no LPA or Ki16425. (*n* = 6). *p < 0.05 vs. control, **p < 0.01 vs. control, ***p < 0.001 vs. control (**c**) LPA also increases production of other angiogenic and inflammatory cytokines. Data are presented as average subtracted background fluorescence intensity normalized to positive controls.

Because transcriptional activity does not necessarily correspond to functional outcomes, we assessed the ability of LPA-treated, ischemic ASC to attract endothelial cells. To avoid potentially confounding effects of direct cell-cell interactions, we incubated ASC under SD/H for 24 hours in the presence or absence of LPA and collected the conditioned medium for use in a transwell migration assay [23]. Human endothelial colony forming cells (ECFC) were seeded in the top of a modified Boyden chamber, and medium from ASC was placed in the lower compartment to determine chemotactic effect (Figure **3a**). In agreement with our qPCR data, significantly more ECFC migrated toward medium from ASC exposed to LPA under SD/H. Similarly, Ki16425 reversed the effects of LPA conditioning. We also found that ECFC were not attracted to unconditioned medium containing LPA alone, demonstrating that endothelial cells are stimulated directly by ASC-secreted factors. 

### 1.3. LPA stimulates ASC VEGF secretion in 3D fibrin gels

Practical applications for ASC-mediated therapeutic angiogenesis require a biomaterial delivery system to maintain cell localization and survival [5,24]. Therefore, we examined the ability of LPA to maintain its proangiogenic effect in a 3D *in vitro* model. We selected a fibrin hydrogel for our application due to its native role in clots as a provisional matrix for recruited endothelial and immune cells [28]. To replicate an *in vivo* environment as accurately as possible, gels were synthesized with an internal concentration of 25 μM LPA, but no lipid was added to the surrounding culture medium (Figure **3b**). Our 3D results reflect 2D gene expression – specifically, ASC entrapped in fibrin gels containing LPA secrete significantly higher amounts of VEGF into the surrounding medium, while Ki16425 completely abolishes this effect. 

We further examined the secretion of additional factors using an angiogenesis antibody array (Figure **3c**). These data revealed that, in addition to producing higher levels of VEGF, ASC entrapped in LPA-containing gels secrete more angiogenin, another powerful angiogenic factor. Additionally, these cells produced more MCP-1 (*not shown*), TIMP-2, and RANTES, but less TGF-β1, suggesting that the addition of LPA affects the immunomodulatory potency of ASC under SD/H. Again, the addition of Ki16425 reversed the effects of LPA for all cytokines.

### 1.4. Co-delivery of ASC and LPA significantly improves critical limb ischemia recovery

**Figure 4:**
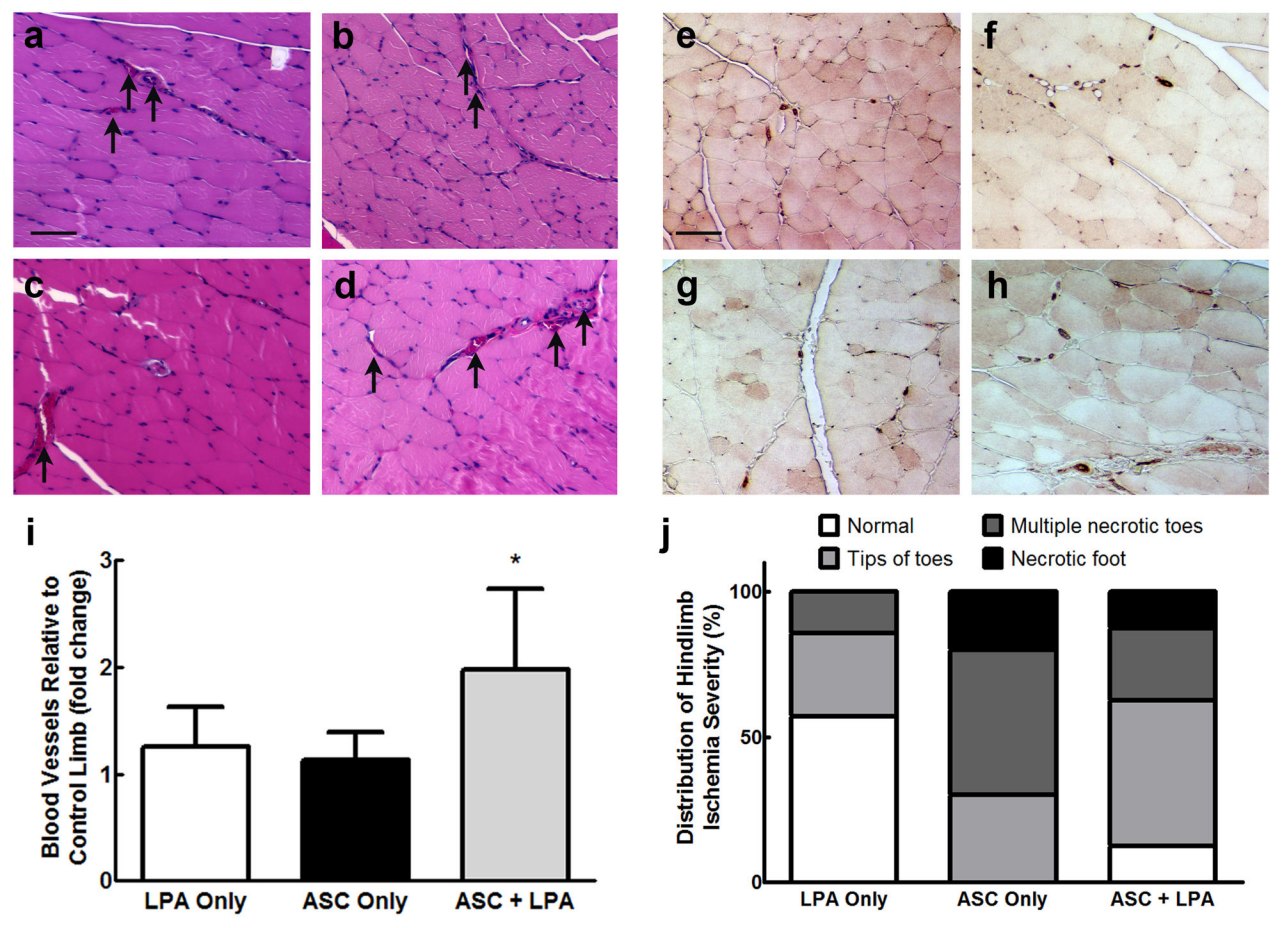
Co-delivery of LPA with ASC in fibrin gels significantly improves angiogenesis in a murine model of critical limb ischemia. Representative H&E stained sections from the quadriceps of (**a**) normal hindlimbs and (**b**) ischemic limbs treated with fibrin gels containing 25 μM LPA, (**c**) ASC, or (**d**) LPA and ASC show the presence of more large vessels in limbs treated with cells and LPA together. Similarly, CD31 staining reveals the formation of larger intramuscular blood vessels in limbs receiving (**h**) both LPA and ASC, while (**e**) normal tissue and defects receiving (**f**) LPA and (**g**) ASC alone have fewer and smaller vessels. (**i**) Blood vessel quantification from H&E stained sections confirms these results (*n=8*). Scale bars represent 100 μm; arrows indicative of vessels with defined lumens and erythrocytes. *p < 0.05 vs. other groups. (**j**) Hindlimbs were visually assessed for severity of toe and foot necrosis in each treatment group.

We applied our findings in a rigorous model of critical limb ischemia using NSG mice by creating a severe, unilateral ischemic defect that leads to limb necrosis if left untreated. We implanted fibrin gels containing LPA, ASC, or both LPA and ASC over the ligated artery site and assessed intramuscular vascularization 2 weeks post-surgery. H&E stained sections of quadriceps distal to the femoral artery ligation site revealed significantly more large blood vessels in limbs treated with gels containing cells and LPA together (Figure **4a-d**). Quantification of these vessels, defined as clear circular structures with a lumen and containing more than one erythrocyte, confirmed this outcome (Figure **4i**). To completely visualize all blood vessels, including microcapillaries, we stained the histological sections for CD31 (Figure **4e-h**). In agreement with H&E results, limbs treated with the combination of ASC and LPA had more numerous vessels (Figure **4i**). Of particular note, mice receiving ASC and LPA lost fewer toes and experienced less severe necrosis than mice receiving ASC alone, although animals treated with LPA only had the least overall limb damage (Figure **4j**). Taken together with our *in vitro* data, these findings demonstrate that the proangiogenic potential of ASC is significantly increased when treated with LPA, and this strategy has great translational potential for use in clinical conditions of vascular disease. 

## Discussion

Therapeutic angiogenesis for treating PVD is frequently pursued through the delivery of recombinant proteins such as VEGF and FGF to promote vascularization. However, these proteins are expensive and suffer from a characteristic, rapid “burst” release profile when entrapped in hydrogels, making sustained delivery challenging and potentially requiring the use of high concentrations that can have undesirable side effects [29,30]. For example, the localized or systemic administration of high concentrations of recombinant VEGF has been implicated in awakening dormant tumor cells or promoting the growth of aberrant blood vessels that are quickly pruned through vascular remodeling [31,32]. Although some groups have pursued the covalent attachment of VEGF to a gel matrix [33], the material-specific nature of the required chemistry limits widespread applicability. As an alternative to protein release, the delivery of self-assembling peptides with proangiogenic molecules provides a method for more sustained presentation [34,35], but such synthetic peptides must be carefully designed for optimal non-immunogenicity and degradation properties for each application. 

Our findings describe a novel method for cell-based angiogenesis that eliminates the need for costly recombinant growth factors. Using LPA, an inexpensive glycerophospholipid (~$10/mg versus ~$4,300/mg for VEGF and $12,000/mg for angiopoietin [36]), we observed synergistically enhanced, functional proangiogenic activity of ASC, a cell population that has a well-described role in supporting endothelial cells and neovascularization. Closely related lipid mediators such as sphingosine-1-phosphate (S1P) [37] have been investigated for their direct angiogenic effects on endothelial progenitor cells [38,39], but less focus has been given to the mural cells that are critical for stabilizing vessel formation and regulating endothelial cell growth [40]. 

Although LPA stimulates VEGF production in rat stromal cells [41] and human cancer cell lines [20] under ischemia, such findings lack translational relevance because of differences in chemokine receptor expression [42] and inter-species inconsistencies in endogenous serum LPA levels. In fact, LPA concentrations in plasma from Wistar rats are three orders of magnitude higher than in FBS or human serum (*data not shown*). Similarly, LPA from ovarian cancer cells increases VEGF secretion in ASC [19], but the use of cell-conditioned media containing a plethora of signaling molecules and biologically variant concentrations of LPA is confounding and thus not ideal for therapeutic angiogenesis.

Our *in vitro* results demonstrate that 25 μM LPA upregulates ASC expression of *VEGF* and *FGF2* under ischemic conditions, and that these increases in transcriptional activity are reflected by actual VEGF secretion. ASC also produced more angiogenin, as well as TIMP-2, a matrix metalloproteinase (MMP) inhibitor. RANTES (CCL5) and MCP-1, both of which recruit monocytes and macrophages to sites of inflammation [43], were secreted at higher levels as well, suggesting that LPA may also have *in vivo* immunomodulatory effects. 

Furthermore, LPA-treated ASC are significantly more effective at recruiting endothelial cells through paracrine signaling. Although LPA alone promotes angiogenesis in a chick chorioallantoic membrane (CAM) model [44], our results specifically show that 25 μM LPA is not chemoattractive to human endothelial cells. Of particular relevance for clinical applications, we successfully showed that ASC respond to LPA in SD/H when incorporated in fibrin gels, without the need for any preconditioning regimens. We also found that LPA_1/3_ inhibitor Ki16425 abolished all LPA-induced effects on ASC. High levels of *LPAR1* expression, coupled with the absence of *LPAR3*, indicate that LPA_1_ mediates the angiogenic signaling of LPA in human ASC. This is consistent with our findings that LPA_1_ is responsible for anti-apoptotic and pro-angiogenic effects of LPA in bone marrow-derived MSC [22].

Most importantly, we demonstrated that LPA maintains its effects on ASC *in vivo*. We implanted fibrin-entrapped human ASC in a severe model of critical limb ischemia that results in rapid limb loss if untreated. The co-delivery of ASC and 25 μM LPA improved functional outcome and significantly increased blood vessel formation in the defect area within 2 weeks compared to ASC alone, highlighting the exciting potential of this treatment method. Although limbs treated with LPA alone showed less overall limb loss, there was no corresponding increase in blood vessel numbers at two weeks. Recent work with S1P, a closely related bioactive lipid that also signals through G-protein coupled receptors [45], demonstrated that S1P recruits anti-inflammatory monocytes and M2 macrophages to blood vessels in inflamed and ischemic tissue [46]. These findings, coupled with the LPA-mediated increase in MCP-1 and RANTES, suggest that LPA alone may reduce initial inflammation *in situ*, thus preserving more tissue at an early stage. However, new blood vessel formation may be limited without the additional proangiogenic factors secreted by LPA-treated ASC. 

Notably, the ability to synthesize fibrin gels containing cells and LPA at the point of care lends itself readily to clinical situations. Our treatment would be particularly effective if combined with methods currently in development for rapidly isolating and concentrating the stromal vascular fraction from adipose tissue [47,48]. The ability to isolate and entrap autologous ASC in LPA-containing fibrin gels and implant them in a defect site during the course of a single surgery would represent a significant advance over previous *in vivo* cardiac studies that require rat MSCs to be preconditioned *in vitro* before injection [41]. Overall, our results have broad translational applications, including the possible treatment of severe limb ischemia by co-delivery of LPA and autologous ASC. 

## References

[1] Peach G, Griffin M, Jones KG, Thompson MM, Hinchliffe RJ (2012) Diagnosis and management of peripheral arterial disease. BMJ 345: e5208. doi:10.1136/bmj.e5208. PubMed: 22893640. 22893640

[2] Shammas NW (2007) Epidemiology, classification, and modifiable risk factors of peripheral arterial disease. Vasc Health Risk Manag 3: 229-234. doi:10.2147/vhrm.2007.3.2.229. PubMed: 17580733. 17580733 PMC1994028

[3] Silva EA, Mooney DJ (2007) Spatiotemporal control of vascular endothelial growth factor delivery from injectable hydrogels enhances angiogenesis. J Thromb Haemost 5: 590-598. doi:10.1111/j.1538-7836.2007.02386.x. PubMed: 17229044. 17229044

[4] Ferrara N, Kerbel RS (2005) Angiogenesis as a therapeutic target. Nature 438: 967-974. doi:10.1038/nature04483. PubMed: 16355214. 16355214

[5] Silva EA, Kim ES, Kong HJ, Mooney DJ (2008) Material-based deployment enhances efficacy of endothelial progenitor cells. Proc Natl Acad Sci U S A 105: 14347-14352. doi:10.1073/pnas.0803873105. PubMed: 18794520. 18794520 PMC2567164

[6] Nakagami H, Morishita R, Maeda K, Kikuchi Y, Ogihara T et al. (2006) Adipose tissue-derived stromal cells as a novel option for regenerative cell therapy. J Atheroscler Thromb 13: 77-81. doi:10.5551/jat.13.77. PubMed: 16733294. 16733294

[7] Katare R, Riu F, Rowlinson J, Lewis A, Holden R et al. (2013) Perivascular Delivery of Encapsulated Mesenchymal Stem Cells Improves Postischemic Angiogenesis Via Paracrine Activation of VEGF-A. Arterioscler Thromb Vasc Biol 33: 1872–1880. PubMed: 23766261. 23766261 10.1161/ATVBAHA.113.301217

[8] Cheung WK, Working DM, Galuppo LD, Leach JK (2010) Osteogenic comparison of expanded and uncultured adipose stromal cells. Cytotherapy 12: 554-562. doi:10.3109/14653241003709694. PubMed: 20370353. 20370353

[9] Bunnell BA, Flaat M, Gagliardi C, Patel B, Ripoll C (2008) Adipose-derived stem cells: isolation, expansion and differentiation. Methods 45: 115-120. doi:10.1016/j.ymeth.2008.03.006. PubMed: 18593609. 18593609 PMC3668445

[10] Cao Y (2010) Adipose tissue angiogenesis as a therapeutic target for obesity and metabolic diseases. Nat Rev Drug Discov 9: 107-115. doi:10.1038/nrd3055. PubMed: 20118961. 20118961

[11] de Villiers JA, Houreld N, Abrahamse H (2009) Adipose derived stem cells and smooth muscle cells: implications for regenerative medicine. Stem Cell Rev 5: 256-265. doi:10.1007/s12015-009-9084-y. 19669954

[12] He J, Genetos DC, Yellowley CE, Leach JK (2010) Oxygen tension differentially influences osteogenic differentiation of human adipose stem cells in 2D and 3D cultures. J Cell Biochem 110: 87-96. PubMed: 20213746. 20213746 10.1002/jcb.22514

[13] Tigyi G (2010) Aiming drug discovery at lysophosphatidic acid targets. Br J Pharmacol 161: 241-270. doi:10.1111/j.1476-5381.2010.00815.x. PubMed: 20735414. 20735414 PMC2989581

[14] Moolenaar WH (1995) Lysophosphatidic acid, a multifunctional phospholipid messenger. J Biol Chem 270: 12949-12952. doi:10.1074/jbc.270.22.12949. PubMed: 7768880. 7768880

[15] Chen J, Baydoun AR, Xu R, Deng L, Liu X et al. (2008) Lysophosphatidic acid protects mesenchymal stem cells against hypoxia and serum deprivation-induced apoptosis. Stem Cells 26: 135-145. doi:10.1634/stemcells.2007-0098. PubMed: 17932426. 17932426

[16] Masiello LM, Fotos JS, Galileo DS, Karin NJ (2006) Lysophosphatidic acid induces chemotaxis in MC3T3-E1 osteoblastic cells. Bone 39: 72-82. doi:10.1016/j.bone.2005.12.013. PubMed: 16487757. 16487757

[17] Mansell JP, Blackburn J (2013) Lysophosphatidic acid, human osteoblast formation, maturation and the role of 1alpha,25-Dihydroxyvitamin D3 (calcitriol). Biochim Biophys Acta 1831: 105-108. 22561288 10.1016/j.bbalip.2012.04.005

[18] Lee MJ, Jeon ES, Lee JS, Cho M, Suh DS et al. (2008) Lysophosphatidic acid in malignant ascites stimulates migration of human mesenchymal stem cells. J Cell Biochem 104: 499-510. doi:10.1002/jcb.21641. PubMed: 18027882. 18027882

[19] Jeon ES, Heo SC, Lee IH, Choi YJ, Park JH et al. (2010) Ovarian cancer-derived lysophosphatidic acid stimulates secretion of VEGF and stromal cell-derived factor-1 alpha from human mesenchymal stem cells. Exp Mol Med 42: 280-293. doi:10.3858/emm.2010.42.4.027. PubMed: 20177148. 20177148 PMC2859327

[20] Park SY, Jeong KJ, Lee J, Yoon DS, Choi WS et al. (2007) Hypoxia enhances LPA-induced HIF-1alpha and VEGF expression: their inhibition by resveratrol. Cancer Lett 258: 63-69. doi:10.1016/j.canlet.2007.08.011. PubMed: 17919812. 17919812

[21] Lee J, Park SY, Lee EK, Park CG, Chung HC et al. (2006) Activation of hypoxia-inducible factor-1alpha is necessary for lysophosphatidic acid-induced vascular endothelial growth factor expression. Clin Cancer Res 12: 6351-6358. doi:10.1158/1078-0432.CCR-06-1252. PubMed: 17085645. 17085645

[22] Binder BY, Genetos DC, Leach JK (2013) Lysophosphatidic acid protects human mesenchymal stromal cells from differentiation-dependent vulnerability to apoptosis. Tissue Eng Part A. In press PubMed: 24131310. 10.1089/ten.tea.2013.0487 PMC3993074 24131310

[23] Hoch AI, Binder BY, Genetos DC, Leach JK (2012) Differentiation-dependent secretion of proangiogenic factors by mesenchymal stem cells. PLOS ONE 7: e35579. doi:10.1371/journal.pone.0035579. PubMed: 22536411. 22536411 PMC3334972

[24] Davis HE, Miller SL, Case EM, Leach JK (2011) Supplementation of fibrin gels with sodium chloride enhances physical properties and ensuing osteogenic response. Acta Biomater 7: 691-699. doi:10.1016/j.actbio.2010.09.007. PubMed: 20837168. 20837168

[25] Murphy KC, Leach JK (2012) A reproducible, high throughput method for fabricating fibrin gels. BMC Res Notes 5: 423. doi:10.1186/1756-0500-5-423. PubMed: 22873708. 22873708 PMC3492004

[26] Fierro FA, Kalomoiris S, Sondergaard CS, Nolta JA (2011) Effects on proliferation and differentiation of multipotent bone marrow stromal cells engineered to express growth factors for combined cell and gene therapy. Stem Cells 29: 1727-1737. doi:10.1002/stem.720. PubMed: 21898687. 21898687 PMC3784258

[27] He J, Genetos DC, Leach JK (2010) Osteogenesis and trophic factor secretion are influenced by the composition of hydroxyapatite/poly(lactide-co-glycolide) composite scaffolds. Tissue Eng Part A 16: 127-137. doi:10.1089/ten.tea.2009.0255. PubMed: 19642853. 19642853 PMC2811060

[28] van Hinsbergh VW, Collen A, Koolwijk P (2001) Role of fibrin matrix in angiogenesis. Ann N Y Acad Sci 936: 426-437. PubMed: 11460496. 11460496 10.1111/j.1749-6632.2001.tb03526.x

[29] Davis HE, Leach JK (2011) Designing bioactive delivery systems for tissue regeneration. Ann Biomed Eng 39: 1-13. doi:10.1007/s10439-010-0135-y. PubMed: 20676773. 20676773 PMC3010216

[30] Sun Q, Silva EA, Wang A, Fritton JC, Mooney DJ et al. (2010) Sustained release of multiple growth factors from injectable polymeric system as a novel therapeutic approach towards angiogenesis. Pharm Res 27: 264-271. doi:10.1007/s11095-009-0014-0. PubMed: 19953308. 19953308 PMC2812420

[31] Neufeld G, Cohen T, Gengrinovitch S, Poltorak Z (1999) Vascular endothelial growth factor (VEGF) and its receptors. FASEB J 13: 9-22. PubMed: 9872925. 9872925

[32] Saranadasa M, Wang ES (2011) Vascular endothelial growth factor inhibition: conflicting roles in tumor growth. Cytokine 53: 115-129. doi:10.1016/j.cyto.2010.06.012. PubMed: 20708948. 20708948

[33] Zisch AH, Lutolf MP, Ehrbar M, Raeber GP, Rizzi SC et al. (2003) Cell-demanded release of VEGF from synthetic, biointeractive cell ingrowth matrices for vascularized tissue growth. FASEB J 17: 2260-2262. PubMed: 14563693. 14563693 10.1096/fj.02-1041fje

[34] Guo HD, Cui GH, Yang JJ, Wang C, Zhu J et al. (2012) Sustained delivery of VEGF from designer self-assembling peptides improves cardiac function after myocardial infarction. Biochem Biophys Res Commun 424: 105-111. doi:10.1016/j.bbrc.2012.06.080. PubMed: 22732415. 22732415

[35] Kim JH, Jung Y, Kim SH, Sun K, Choi J et al. (2011) The enhancement of mature vessel formation and cardiac function in infarcted hearts using dual growth factor delivery with self-assembling peptides. Biomaterials 32: 6080-6088. PubMed: 21636123. 21636123 10.1016/j.biomaterials.2011.05.003

[36] Systems RD (2012). R&D Systems Annual Catalog.

[37] Spiegel S, Milstien S (2003) Sphingosine-1-phosphate: an enigmatic signalling lipid. Nat Rev Mol Cell Biol 4: 397-407. doi:10.1038/nrm1103. PubMed: 12728273. 12728273

[38] Walter DH, Rochwalsky U, Reinhold J, Seeger F, Aicher A et al. (2007) Sphingosine-1-phosphate stimulates the functional capacity of progenitor cells by activation of the CXCR4-dependent signaling pathway via the S1P3 receptor. Arterioscler Thromb Vasc Biol 27: 275-282. PubMed: 17158356. 17158356 10.1161/01.ATV.0000254669.12675.70

[39] Ozaki H, Hla T, Lee MJ (2003) Sphingosine-1-phosphate signaling in endothelial activation. J Atheroscler Thromb 10: 125-131. doi:10.5551/jat.10.125. PubMed: 14564080. 14564080

[40] Armulik A, Abramsson A, Betsholtz C (2005) Endothelial/pericyte interactions. Circ Res 97: 512-523. doi:10.1161/01.RES.0000182903.16652.d7. PubMed: 16166562. 16166562

[41] Liu X, Hou J, Shi L, Chen J, Sang J et al. (2009) Lysophosphatidic acid protects mesenchymal stem cells against ischemia-induced apoptosis in vivo. Stem Cells Dev 18: 947-954. doi:10.1089/scd.2008.0352. PubMed: 19193014. 19193014

[42] Chamberlain G, Wright K, Rot A, Ashton B, Middleton J (2008) Murine mesenchymal stem cells exhibit a restricted repertoire of functional chemokine receptors: comparison with human. PLOS ONE 3: e2934. doi:10.1371/journal.pone.0002934. PubMed: 18698345. 18698345 PMC2488395

[43] Deshmane SL, Kremlev S, Amini S, Sawaya BE (2009) Monocyte chemoattractant protein-1 (MCP-1): an overview. J Interferon Cytokine Res 29: 313-326. doi:10.1089/jir.2008.0027. PubMed: 19441883. 19441883 PMC2755091

[44] Rivera-Lopez CM, Tucker AL, Lynch KR (2008) Lysophosphatidic acid (LPA) and angiogenesis. Angiogenesis 11: 301-310. doi:10.1007/s10456-008-9113-5. PubMed: 18504643. 18504643 PMC2677190

[45] Pyne NJ, Pyne S (2008) Sphingosine 1-phosphate, lysophosphatidic acid and growth factor signaling and termination. Biochim Biophys Acta 1781: 467-476. doi:10.1016/j.bbalip.2008.05.004. PubMed: 18558100. 18558100

[46] Awojoodu AO, Ogle ME, Sefcik LS, Bowers DT, Martin K et al. (2013) Sphingosine 1-phosphate receptor 3 regulates recruitment of anti-inflammatory monocytes to microvessels during implant arteriogenesis. Proc Natl Acad Sci U S A 110: 13785-13790. doi:10.1073/pnas.1221309110. PubMed: 23918395. 23918395 PMC3752259

[47] Lin K, Matsubara Y, Masuda Y, Togashi K, Ohno T et al. (2008) Characterization of adipose tissue-derived cells isolated with the Celution system. Cytotherapy 10: 417-426. doi:10.1080/14653240801982979. PubMed: 18574774. 18574774

[48] Riordan NH, Ichim TE, Min WP, Wang H, Solano F et al. (2009) Non-expanded adipose stromal vascular fraction cell therapy for multiple sclerosis. J Transl Med 7: 29. doi:10.1186/1479-5876-7-29. PubMed: 19393041. 19393041 PMC2679713

